# Virus-Mediated Overexpression of Two Allelic Protein Fragments Elicits Drastically Different Responses in Soybean

**DOI:** 10.3390/v18040419

**Published:** 2026-03-29

**Authors:** Seung Hyun Yang, Anna Favalon, Junping Han, Camila Perdoncini Carvalho, Leah McHale, Anne Dorrance, Feng Qu

**Affiliations:** 1 Department of Plant Pathology, The Ohio State University, Wooster, OH 44691, USA; seunghyunlee252@gmail.com (S.H.Y.); annafavalon1@gmail.com (A.F.); han.393@osu.edu (J.H.); perdoncinicarvalho.1@osu.edu (C.P.C.); dorrance.1@osu.edu (A.D.); 2 Department of Horticulture and Crop Science, The Ohio State University, Columbus, OH 43210, USA; mchale.21@osu.edu

**Keywords:** cowpea severe mosaic virus, virus-induced gene silencing, virus-mediated protein expression, soybean, protein kinase, quantitative resistance, hypersensitive response, plant functional genomics

## Abstract

Soybean (*Glycine max*) is relatively recalcitrant to genetic manipulations; hence, it is often interrogated with transient means such as virus-induced gene silencing (VIGS). We earlier modified cowpea severe mosaic virus (CPSMV) to develop a soybean-friendly VIGS system referred to as QUIN-FZ. Here we report additional calibrations of this system. We enhanced the intra-bacterial stability of plasmid QUIN, which contained a CPSMV RNA1 cDNA embedded with four introns, by adding a fifth intron, resulting in PENTIN. We separately upgraded the plasmid FZ, which contained a modified CPSMV RNA2 cDNA with a cloning site in the middle of the viral polyprotein, by creating another cloning site within the 3′ untranslated region, leading to ZY. We next used the new PENTIN-ZY system to investigate a putative soybean protein kinase designated QL18. Virus-mediated overexpression of two allelic, 147-amino-acid (aa) protein fragments, derived from two different QL18 orthologs, elicited drastically different responses in soybeans. While the fragment derived from soybean accession OX20-8 prevented the cognate virus from infecting top young leaves in at least 50% of inoculated seedlings, its allelic counterpart derived from soybean accession PI427105B elicited apical necrosis in 100% of soybean seedlings. By examining progeny viruses as well as viruses encoding chimeras of the two fragments, we identified more than a dozen mutations that abrogated these unique phenotypes. Our findings establish the PENTIN-ZY system as a versatile tool for overexpressing small proteins and protein fragments, accelerating their functional characterization.

## 1. Introduction

With increasing availability of full genome sequences of diverse crop plants [1,2,3], we are now faced with the challenge of understanding the functions of individual plant genes, especially those playing important roles in the yield and quality of agricultural products, and those conferring protection against pathogens, insects, and abiotic stresses. Functional genomics investigations in model plants mostly take the reverse genetics approach that entails transgenic knock-out or over-expression of genes-of-interest (GOIs), followed by analyses of phenotypic consequences of these manipulations [4]. However, adoption of reverse genetics approaches in many agricultural crops, including soybean (*Glycine max*), has encountered many hurdles. First, transgenic ablation or over-expression of GOIs is less straightforward, as soybean is hard to transform, and the process of obtaining transgenic seed is time-consuming and laborious [5]. Also, due to the low efficiency and genotype-dependence of soybean transformation, genome-wide mutant libraries widely available for Arabidopsis and rice (e.g., T-DNA insertion libraries) were attempted in very few soybean genotypes, and with low levels of saturation. Finally, in contrast to model plants that are relatively small in size and easy to grow year-round in greenhouses, soybean plants are large in size, and usually require open fields for propagation of transgenic stocks [6,7]. Thus, the classic reverse genetic approach is inefficient and impractical for interrogating soybean genes, and alternative approaches are needed to accelerate functional characterization of soybean genes of significant economic values.

Virus-induced gene silencing (VIGS) is an alternative functional genomics tool that does not involve generation of transgenic plants. Instead, it uses a virus, most commonly a plus strand (+) RNA virus, that is modified to carry a partial fragment of a plant GOI [8]. Infection of the same plant species with the modified virus triggers a plant defense response known as RNA silencing or RNA interference (RNAi) that targets viral RNA for degradation in a highly sequence-specific manner [8]. Since the modified virus carries a host gene fragment, the specific RNA silencing response also generates small interfering RNAs (siRNAs) that target the cognate host gene mRNA for degradation, causing down-regulation, or silencing, of this gene.

Several viruses have been developed into VIGS vectors for soybean use. A bean pod mottle virus (BPMV)-based VIGS vector was developed almost 20 years ago and widely used by the soybean research community [9,10,11,12]. Another VIGS vector based on apple latent spherical virus (ALSV) has also been successfully used in select soybean varieties [13,14,15,16,17]. However, both vectors have shortcomings. The earliest BPMV VIGS vector relied on in vitro transcribed viral RNAs that must be capped at the 5′ end to gain infectivity [9]. Such capped transcripts are expensive to prepare. Newer BPMV vectors depended on delivery with the sophisticated particle bombardment equipment [10,18]. On the other hand, the ALSV-based VIGS vector can only infect a few soybean accessions with sufficient efficacies, greatly limiting its usefulness [14]. In addition to BPMV and ALSV, a soybean-infecting tobacco ringspot virus (TRSV) isolate has also been modified into a VIGS vector and used to silence soybean genes [19].

**Figure 1:**
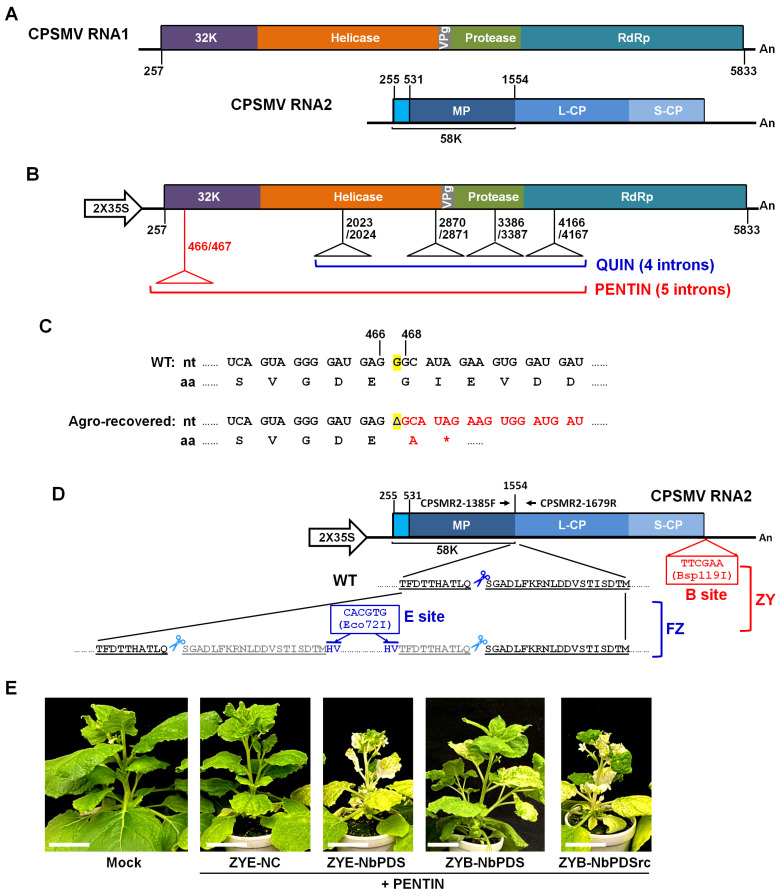
Optimization of the CPSMV-based vector system. (**A**) Schematic representation of CPSMV RNA1 and 2. (**B**) The QUIN (4 introns) and PENTIN (5 introns) constructs harboring the modified CPSMV RNA1 cDNA. 2X35S: the duplicated 35S promoter of cauliflower mosaic virus. (**C**). Location of the single-nt deletion (G at position 467) within CPSMV RNA1 cDNA responsible for alleviation of toxicity but also loss of plant infectivity. Note this deletion caused the reading frame of RNA1 polyprotein to shift into a new frame that, after just one GCA codon, encounters a UAG stop codon. (**D**) The CPSMV RNA2-derived FZ vector with the intra-polyprotein Eco72I cloning site, and the new ZY vector with both Eco72I site and the new Bsp119I cloning site within 3′ UTR. (**E**) Infectivity of PENTIN and ZY constructs as tested in *N. benthamiana* plants, and the relative potency of ZYE and ZYB-based VIGS constructs at inducing NbPDS silencing. The images were collected at 3 weeks post agro-infiltration. Scale bars = 5 cm.

We reported earlier the successful engineering of cowpea severe mosaic virus (CPSMV) into a vector system suitable for VIGS in *Nicotiana benthamiana* and soybean [20]. CPSMV is a (+) RNA virus belonging to the virus family *Secoviridae*, genus *Comovirus* [21,22], and a close relative of BPMV. The CPSMV genome comprises two (+) RNAs—RNA1 and RNA2 (Figure 1A). RNA1 encodes a single polyprotein that, upon translation, is processed into multiple functional proteins by a protease (Pro) embedded in the polyprotein (Figure 1A). These mature proteins, including a 32K protein (named after the approximate size of this protein—32 kilodaltons), a helicase, a small genome-linked protein (VPg), Pro, and a viral RNA-dependent RNA polymerase (RdRp), are needed for the replication of both RNA1 and 2 (Figure 1A). By contrast, CPSMV RNA2 encodes two nearly identical polyproteins by adopting two different translational start sites (Figure 1A). The larger polyprotein starts at the nucleotide (nt) position 255, whereas the smaller one starts at position 531 [20]. Proteolytic processing of these two polyproteins by Pro gives rise to two N-proximal proteins—58K and movement protein (MP), with the former being 92 amino acids (aas) longer at the N-terminus. The 58K protein is likely required for RNA2 replication [18], whereas MP mediates the cell-to-cell movement of this virus. Nevertheless, both RNA2-encoded polyproteins are thought to yield the same capsid protein subunits, known as L-CP and S-CP, both needed for assembly of CPSMV particles (Figure 1A).

In the earlier study, we obtained full-length cDNAs of CPSMV RNA1 and 2, and placed them under control of a strong promoter (2X35S of cauliflower mosaic virus) in the binary plasmid pAI101 [20]. We further modified the RNA1 cDNA by incorporating four introns that dramatically stabilized the resulting QUIN construct in both *Escherichia coli* and *Agrobacterium tumefaciens*. We additionally modified RNA2 to embed an Eco72I site with appropriate contexts to accommodate the insertion of GOI fragments, and named the modified RNA2 construct FZ. This QUIN-FZ VIGS system facilitated robust VIGS in *N. benthamiana* and soybean [20]. Importantly, derivatives of FZ with GOI inserts can be easily propagated into virions in Agrobacterium (agro)-infiltrated *N. benthamiana* plants, whose leaf tissues could then be used to inoculate soybeans. This virus propagation procedure obviated the need for sophisticated equipment, was thus easily adoptable by most soybean research laboratories.

However, subsequent uses of the QUIN-FZ system revealed a few flaws. First, the QUIN construct harboring CPSMV RNA1 cDNA, while propagating normally in *E. coli* cells, caused Agrobacterium cells to multiply very slowly, yielding tiny colonies that were visible only after 4-day incubation at 28 °C. More frustratingly, rare Agrobacterial colonies that were visible after just 2-day incubation would fail to initiate CPSMV infections in *N. benthamiana.* This suggests that in these rare colonies spontaneous mutation(s) might have emerged in QUIN that alleviated the toxicity of RNA1 cDNA in Agrobacteria, but also abolished its infectivity in plants. Thus, the RNA1 cDNA in QUIN needed further modifications to mitigate its toxicity in Agrobacterium cells while maintaining its infectivity in plants. In addition, the FZ construct harboring modified CPSMV RNA2 cDNA also required further optimization. Specifically, the location of the Eco72I insertion site in FZ, sitting between MP and L-CP coding sequences, obligates the insert to encode an undisrupted protein fragment in-frame with MP and L-CP, hence severely constraining the insert sizes and orientation.

The current study addresses these challenges by making additional modifications in both QUIN and FZ, leading to PENTIN and ZY, respectively. The updated PENTIN plasmid grows robustly in both *E. coli* and Agrobacteria, and no longer incurs infectivity-defeating mutations. Additionally, the updated ZY construct permits GOI inserts at two different sites, and both sites if needed. Thus, this new PENTIN-ZY system should encourage more widespread adoption of this functional genomics tool. We further used the upgraded PENTIN-ZY system to overexpress two highly similar protein fragments derived from two allelic protein kinases of soybean associated with susceptibility and quantitative resistance to *Phytophthora sojae* [23,24], and examined the dramatically different phenotypic consequences of such overexpression. These findings may offer insights into the role of the underlying protein kinases.

## 2. Materials and Methods

### 2.1. Recombinant DNA Constructs

The constructs QUIN and PENTIN are both derivatives of the binary vector pAI101 [18,25,26]. The construction of QUIN, which harbored a modified CPSMV RNA1 cDNA with four plant-derived introns, has been described in an earlier report [20]. The new PENTIN construct was derived from QUIN by inserting a fifth intron between nt positions 466 and 467 of CPSMV RNA1. This new intron was brought in with a 675-nt synthesized DNA fragment (Integrated DNA Technologies, Coralville, IA, USA) that replaced the region of RNA1 cDNA flanked by the AflII and BbvC1 sites. The sequence of the synthesized DNA fragment is provided in Supplemental Table S1. The cloning was carried out with the NEBuilder Gibson Assembly kit (New England Biolabs, Ipswich, MA, USA). The resulting PENTIN construct was verified with Sanger sequencing.

Similarly, both FZ and ZY were pAI101 derivatives harboring CPSMV RNA2 cDNA. The FZ construct has been described earlier [20]. ZY was generated by introducing a new Bsp119I insertion site within the 3′ UTR of CPSMV RNA2, 9-nt downstream of the stop codon of RNA2-encoded polyprotein. The specific manipulation entailed a 786-bp synthesized DNA fragment (ZY-B-NbTOR in Supplemental Table S1) that replaced the Bsp119I-HpaI fragment of RNA2 cDNA. This synthesized fragment contained two synonymous mutations that abolished the original Bsp119I site (TTCGAA-to-TTTGAG), and a 300-bp insert of an *N. benthamiana* gene (NbTOR) sandwiched between two newly created Bsp119I sites. It was integrated into FZ using the NEBuilder Gibson Assembly kit. The resulting ZY construct was verified with Sanger sequencing. Additional VIGS constructs were assembled by inserting various synthesized DNA fragments into the Eco72I site or the Bsp119I site. The names of constructs with inserts at Eco72I and Bsp119I sites have the ZYE- and ZYB- prefixes, respectively.

### 2.2. Agrobacterium Infiltration (Agro-Infiltration) of N. benthamiana Leaves

All constructs subject to infectivity tests were transformed into electrocompetent *A. tumefaciens* strain C58C1 via electroporation using the AGR setting on the Bio-Rad Micropulser Electroporator [26,27]. Briefly, 5 µL of the plasmid DNA was mixed with 40 µL electro-competent Agrobacterium cells and maintained on ice until electroporation. After electroporation, 900 µL of Super Optimum Broth (SOB) media was added and the suspension was incubated at 28 °C for one hour. Selection was carried out on solid Terrific Broth (TB) media containing rifampicin, gentamycin, and kanamycin. Successful introduction of the plasmid was confirmed using colony PCR. A single colony confirmed to have the desired plasmid was used to inoculate 3 mL TB liquid media with the same antibiotics, and incubated overnight at 28 °C. The culture was diluted 1:100 with fresh TB liquid media and incubated under the same conditions for another night. The second culture was centrifuged at 4000 rpm for 20 min, and resuspended in agroinfiltration buffer (10 mM MgCl_2_, 10 mM MES, and 100 µM acetosyringone). All suspensions were diluted to OD_600_ = 1 and incubated at 28 °C for 3 h. *Agrobacterium* suspensions were then mixed and introduced into leaves of young *N. bethamiana* plants via a small wound, using a needleless syringe. Upon successful systemic infection, the symptomatic top leaves were harvested at 14 days after agro-infiltration (14 dai), and stored in −80 °C, or used directly for soybean infection.

### 2.3. Infection of Soybean Seedlings with Viruses Pre-Propagated in N. benthamiana

Seed of OX20-8, Williams (Wm-0) and Williams 79 (Wm-79) accessions were sowed in 32-ounce Styrofoam cups filled with a 1:1 mix of Metro-Mix 360 and greenhouse soil. Each pot is usually sowed with at least 25 seed to ensure germination of at least 10 healthy seedlings. As soon as the earliest true leaves became mostly expanded (with the angle between two halves of a leaf widening to at least 120 degrees), these leaves were rub-inoculated with saps of systemically infected *N. benthamiana* leaves. The virus-inoculated soybean plants were kept in a Conviron growth chamber (25 °C, 14-h day) for up to 28 days.

### 2.4. Evaluation of the Levels of Viral RNA and Plant Gene mRNA with RT-PCR

Total RNA was extracted from systemically infected leaves of *N. benthamiana* or soybean using the Zymo RNA Extraction kit (Irvine, CA, USA). A DNase treatment step was always included to eliminate potential contamination of plant genomic DNA. The quality of the RNA samples was assessed first with a NanoDrop spectrometer, followed by agarose gel electrophoresis to determine RNA integrity. For RT-PCR, an equal amount (usually 1 µg) of RNA was used for all samples in the same experimental group, and the 1st strand cDNA synthesis was primed with a poly-dT primer. At the PCR step, a pair of soybean actin 4 (GmACT4) primers were used to amplify a cDNA fragment that serves as a control to ensure that cDNA synthesis in all samples was initiated with an equivalent amount of mRNA. Various primer pairs (Supplemental Table S2) that amplify soybean GOI cDNAs were used to assess the levels of the corresponding mRNAs.

## 3. Results and Discussion

### 3.1. Neutralizing the Toxicity of CPSMV RNA1 cDNA in Agrobacterium Cells with a Fifth Intron

To develop a CPSMV-based VIGS system, we previously generated a binary plasmid, designated QUIN, in which the CPSMV RNA1 cDNA was modified to contain four introns at various positions [20] (Figure 1B). These four introns were needed because a construct containing the intron-free CPSMV RNA1 cDNA was highly toxic to both *E. coli* and Agrobacteria, yielding extremely small bacterial colonies and very low plasmid DNA titers [20]. By contrast, *E. coli* cells transformed with QUIN grew to levels similar to those transformed with the backbone plasmid pAI101 [18].

Nevertheless, Agrobacterium cells transformed with QUIN still grew slowly, frequently needing four days to yield clearly discernable colonies. Moreover, frequent uses of QUIN by us and others revealed another frustrating issue: plates streaked with the QUIN-transformed Agrobacterium cells occasionally produced larger colonies that were visible with a two-day incubation at 28 °C. However, these larger colonies mostly failed in agro-infiltration-mediated plant infections. To resolve this issue, we chose nine such colonies for further analyses. We purified plasmids from these colonies and subjected the plasmids to Sanger sequencing to determine whether these plasmids incurred any mutations within the CPSMV RNA1 cDNA. Surprisingly, we found that all nine of the plasmids contained the same single-nucleotide (nt) deletion within the triple-G stretch spanning positions 466 and 468 of RNA1 cDNA (Figure 1C). As illustrated in Figure 1C, such a deletion would have caused the reading frame of RNA1-coded polyprotein to shift into a new frame that stops immediately with a UAG stop codon. Thus, this deletion mutation alone accounted for the loss of infectivity of the mutated CPSMV RNA1.

This finding also suggested that this portion of the polyprotein (32K) was likely translated in Agrobacterium cells, and the translated protein in turn suppressed Agrobacteria growth. To address this issue, we further modified the RNA1 cDNA by inserting a fifth intron between positions 466 and 467 (see Materials and Methods, and Supplemental Table S1, for details). This modification led to a new RNA1 cDNA construct, referred to as PENTIN (Figure 1B). Subsequent experiments verified that Agrobacterium cells harboring PENTIN grew as rapidly as those harboring an insert-free pAI101 backbone plasmid. Furthermore, when combined with another plasmid containing a modified RNA2 cDNA harboring a negative control insert (ZYE-NC) in agro-infiltrations, PENTIN caused robust infections of *N. benthamiana* plants in a highly reproducible manner (Figure 1E). Thus, PENTIN is a superior provider of CPSMV RNA1 than QUIN. We thus used PENTIN to replace QUIN in subsequent VIGS experiments.

### 3.2. Upgrading FZ by Adding a Second Cloning Site

The original FZ construct contained a CPSMV RNA2 cDNA that was modified at the protease cleavage site between MP and L-CP (Figure 1D). This specific modification duplicated the amino acid (aa) residues flanking the cleavage site, giving rise to two identical cleavage sites in close vicinity (Figure 1D). An Eco72I site (CACGTG) was then integrated between these two cleavage sites to permit insertion of GOI fragments while keeping the MP and L-CP coding regions intact. This design strategy was also used by others to create BPMV and ALSV-based VIGS vectors [9,13].

One limitation of the FZ vector is that the GOI insert must encode a non-stopping polypeptide in the same reading frame as the RNA2-encoded polyprotein. For protein-coding GOIs this limitation can be relatively easily overcome by simply extracting a portion of the GOI’s own reading frame, and inserting it into FZ in the sense orientation. However, if a frame-shifted or antisense-oriented insert is desired, the insert must be carefully inspected to eliminate stop codons and to ensure continuity with the flanking MP and L-CP frames. To ease this issue, we have tested several positions within the 3′ untranslated region (UTR) of CPSMV RNA2 for a location that accommodated the insertion of silencing-inducing fragments with minimal adverse impact on viral infectivity. These efforts identified the site between 11th and 12th nt downstream of the RNA2 polyprotein stop codon, into which two nt (CG) were inserted to create a new Bsp119I site (TTCGAA) (Figure 1D; Supplemental Table S1). Moreover, insertion of a 300-nt fragment of an *N. benthamiana* gene (NbTOR; Supplemental Table S1) at the new Bsp119I site had negligible impact on viral infectivity. We thus combined this new modification with the existing modifications in FZ, and named the new construct ZY (Figure 1D). Accordingly, constructs with inserts at the Eco72I site were renamed with the ZYE prefix, whereas those with inserts at the new Bsp119I site are named with the ZYB prefix.

We next compared the ZYE and ZYB constructs for VIGS induction using *NbPDS* as the target gene. Note ZYE-NbPDS and ZYB-NbPDS harbored the same NbPDS fragment. However, the latter caused modestly weaker photobleaching than the former, suggesting that insert placed at the 3′ proximal Bsp119I site was less effective at eliciting VIGS than the more upstream Eco72I site (Figure 1E). Nevertheless, when the same insert was placed in a reverse-complemented (rc, i.e., antisense) orientation, the resulting ZYB-NbPDSrc construct was almost as effective as ZYE-NbPDS at triggering photobleaching. These results indicated that the new ZY construct with two different sites for GOI fragment insertion was a more versatile VIGS vector (Figure 1E).

### 3.3. ZYE Constructs Carrying Different Segments of a Putative Protein Kinase cDNA Elicited Unexpected Responses in Soybean

**Figure 2:**
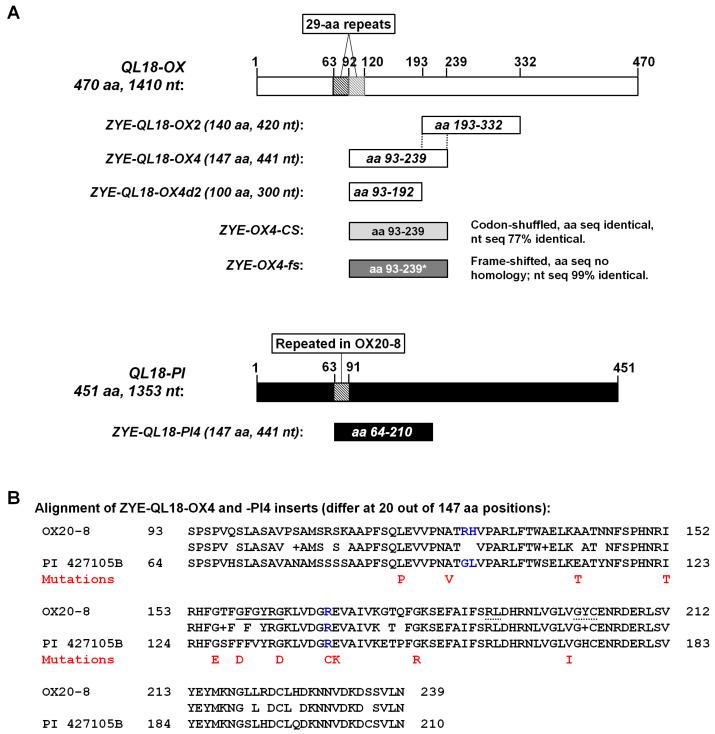
ZYE constructs containing partial fragments of QL18-OX and QL18-PI. (**A**) The full-length coding regions of QL18-OX and QL18-PI are depicted as long unfilled and filled boxes, respectively. The small boxes with hatched lines denote two 29-aa imperfect repeats within QL18-OX. The inserts in various ZYE constructs are shown below the full-length coding regions. (**B**) The deduced aa sequences of the protein fragments encoded by OX4 and PI4 inserts. The red letters denote de novo mutations identified from symptomatic soybean (OX20-8) seedlings inoculated with ZYE-QL18-OX4 and ZYE-OX4-CS.

With the new PENTIN-ZY vector system on hand, we next wanted to interrogate the function of a putative protein kinase of soybean, encoded by Glyma.18G026900. This protein is tentatively referred to as QL18 because earlier research has implicated its alleles in the quantitative resistance to *Phytophthora sojae* [23,24]. Very briefly, the QL18 protein of soybean accession OX20-8 (QL18-OX) was predicted to render soybean more susceptible to *P. sojae*, whereas its allele in accession PI427105B (QL18-PI) was predicted to confer quantitative resistance. Also note that the aa sequence of QL18-OX, but not that of QL18-PI, is identical to other common soybean accessions such as Williams (Wm-0) and Williams 82 (Wm-82). While QL18-OX and QL18-PI differ at many positions, the most striking difference is a 29-aa imperfect repeat present in QL18-OX but absent in QL18-PI (Figure 2A,B; aa residues 92–120 constitute an imperfect repeat of residues 63–91).

**Figure 3:**
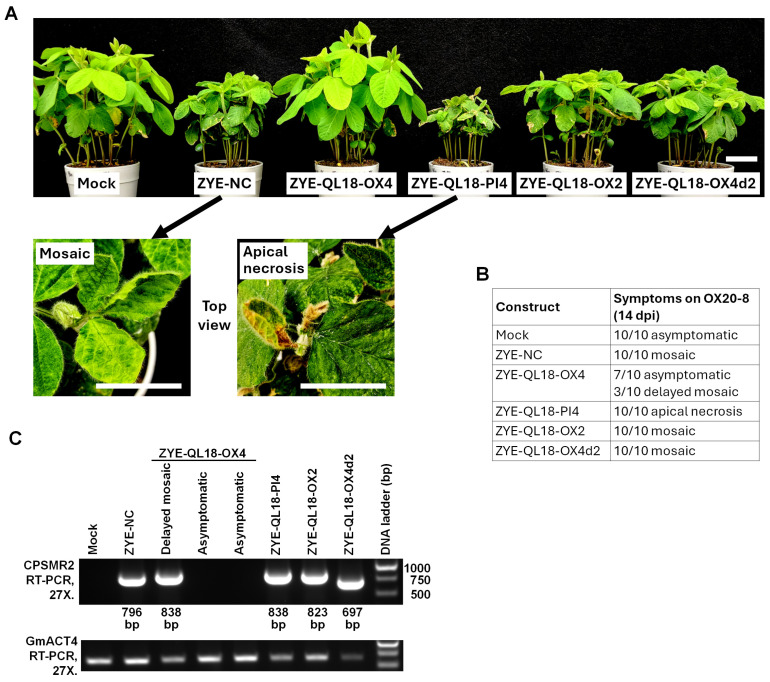
Infection outcomes of OX20-8 seedlings inoculated with ZYE constructs harboring various QL18-derived inserts. (**A**) Systemic symptoms. The top views of ZYE-NC and ZYE-QL18-PI4-infected seedlings were enlarged to show the mosaic and apical necrosis symptoms. Scale bars = 5 cm. (**B**) Numbers of seedlings with varying symptoms from one representative experiment. The experiment was repeated at least three times with similar outcomes. (**C**) RT-PCR results showing the detection of PCR products of expected sizes in symptomatic seedlings, but not in asymptomatic ones.

We first attempted to use ZYE to silence QL18-OX expression through VIGS. To this end, we designed two different constructs, ZYE-QL18-OX2 and ZYE-QL18-OX4 (Figure 2A). These two constructs harbor two partially overlapping fragments of QL18-OX coding sequence, of 420 and 441 nt, respectively, corresponding to aa positions 193–332 (140 aa) and 93–239 (147 aa) (Figure 2A). Both constructs were successfully propagated into infectious viruses in *N. benthamiana*. However, when brought into OX20-8 soybean, they caused drastically different symptoms (Figure 3A). While ZYE-QL18-OX2 elicited systemic mosaic symptoms similar to the negative control (ZYE-NC), ZYE-QL18-OX4 was asymptomatic in more than 50% of inoculated soybean seedlings (Figure 3A,B). Notably, this result was consistently observed in at least four different repeat experiments.

Since the QL18-OX-derived inserts in these two constructs overlapped for 141 nt (47 aa, positions 193–239; Figure 2A), we next generated and tested a ZYE-QL18-OX4d2 construct in which the 141-nt portion of OX4 insert was deleted. As shown in Figure 3A,B, this construct elicited mosaic symptoms resembling that of ZYE-NC and ZYE-QL18-OX2 in OX20-8 seedlings. Thus, although this 141-nt (47 aa) region as part of the OX2 insert did not interfere with viral systemic spread, it nevertheless contributed to the partial infection failure caused by the OX4 insert. We then wondered if the partial failure of systemic infection attributed to the OX4 insert could be overcome by substituting it with its counterpart in PI427105B soybean, designated PI4 (Figure 2 and Figure 3). Unexpectedly, the resulting ZYE-QL18-PI4 construct elicited apical necrosis in 100% of the infected OX20-8 seedlings, leading to stunting and eventual death (Figure 3A,B).

Consistent with lack of systemic symptoms in most OX20-8 seedlings inoculated with ZYE-QL18-OX4, viral RNA of the ZYE-QL18 variants were absent in the asymptomatic seedlings, but detectable in those with symptoms (both mosaic and apical necrosis) (Figure 3C). Unfortunately, we were unable to test these constructs in PI427105B soybean due to insufficient supply of PI427105B seed. Nevertheless, these results demonstrated that in OX20-8 seedlings, ZYE derivatives harboring the allelic OX4 and PI4 inserts elicited drastically different symptoms—the former mostly asymptomatic, whereas the latter apical necrosis.

### 3.4. The Virus-Expressed Protein Fragments, Rather than Their Coding RNAs, Are Responsible for the Differing Symptoms of ZYE-QL18-OX4 and ZYE-QL18-PI4

Though our initial goal was to down-regulate the expression of QL18-OX gene with VIGS, the results presented in Figure 3 could not be easily interpreted as outcomes of VIGS, for at least two reasons. First, the OX2 and OX4 inserts were of similar sizes (Figure 2A), hence would be expected to trigger VIGS to similar extents, yet they induced very different symptoms. Second, the OX4 and PI4 inserts were highly homologous at the nt level, differing at only 26 out of the 441 nt positions, most of them being single-nt substitutions. As a result, they were expected to be similarly potent at triggering VIGS. These considerations led us to hypothesize that virus-mediated expression of OX4 and PI4 protein fragments were responsible for the symptom differences.

**Figure 4:**
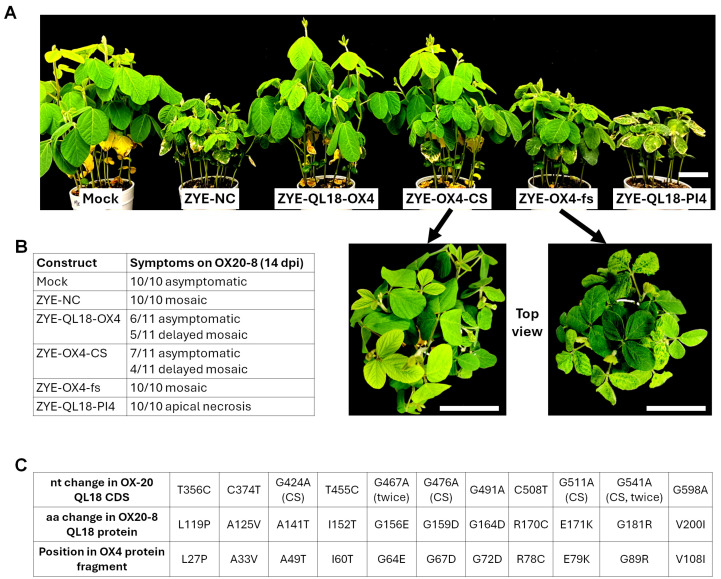
Inability of ZYE-QL18-OX4 to spread systemically in some seedlings is caused by the OX4-encoded partial protein fragment. (**A**) Symptoms of OX20-8 seedlings inoculated with the virus constructs shown. Scale bars = 5 cm. (**B**) Numbers of asymptomatic and symptomatic seedlings in each group. (**C**). De novo mutations identified from viral RNA extracted from symptomatic seedlings inoculated with ZYE-QL18-OX4 and ZYE-OX4-CS.

To test this hypothesis, we generated two more constructs—ZYE-OX4-CS and ZYE-OX4-fs (Figure 2A). In ZYE-OX4-CS, the aa sequence of OX4-encoded protein fragment remained the same, but their codons were shuffled extensively so that the nt level identity with the QL18-OX gene was reduced to 77%. Conversely, in ZYE-OX4-fs, the aa sequence of the original OX4 protein fragment was completely altered with a single-nt insertion after the first codon that caused the reading frame to shift (stop codons within the resulting alternative reading frame were eliminated with nt substitutions); yet the nt sequence was 99% unchanged. As a result, ZYE-OX4-CS would be expected to express the same protein fragment but unable to induce VIGS of QL18-OX, whereas ZYE-OX4-fs would express an entirely different protein, but should still be capable of triggering VIGS of QL18-OX. When brought into OX20-8 seedlings, ZYE-OX4-CS elicited a symptom pattern highly similar to ZYE-QL18-OX4, meaning more than a half of the inoculated seedlings (accession OX20-8) were asymptomatic (Figure 4A,B). By contrast, ZYE-OX4-fs caused typical mosaic symptoms resembling ZYE-NC. Note these experiments were repeated at least three times with similar outcomes. These results lent support to the hypothesis that it was the OX4-encoded protein fragment, rather than VIGS induced by OX4 RNA, that caused the failure of the cognate virus to spread systemically.

### 3.5. Viral RNA Recovered from Symptomatic Seedlings Inoculated with ZYE-QL18-OX4 and ZYE-OX4-CS Contain Amino-Acid-Changing Point Mutations Within Inserts

Given that most OX20-8 seedlings inoculated with ZYE-QL18-OX4 and ZYE-OX4-CS were asymptomatic, the seedlings that did develop symptoms might contain mutant viruses that overcame the defect in systemic spread, probably by incurring mutations in the OX4 and OX4-CS protein fragments. To evaluate this possibility, we have obtained viral RNA samples from 13 symptomatic seedlings—eight from three repeats of ZYE-QL18-OX4 infections, five from two repeats of ZYE-OX4-CS infections. These RNA samples were subsequently subjected to reverse transcription PCR (RT-PCR), and the PCR products were sequenced to identify potential mutations. As summarized in Figure 4C, all 13 PCR products contained point mutations that altered aa residues of the OX4 (and OX4-CS)-encoded protein fragments. Notably, two of the mutations, G156E and G181R, were each recovered twice, suggesting that the underlying wildtype aa residues might play critical roles in restricting the cognate viruses in local leaves.

By mapping the mutations to the alignment in Figure 2B (red font), we found that nine of the eleven mutations, namely L119P, A125V, I152T, G156E, G164D, R170C, E171K, G181R, and V200I (numbering according to the OX20-8 allele), altered aa residues that are conserved in the OX20-8 and PI427105B alleles of QL18. Only two, A141T and G159D, altered non-conserved residues (E112 and F130 in PI427105B allele). Also worth noting was that glycine (G) residues were over-represented among residues undergoing de novo mutations. Among the eleven mutations, four changed a glycine residue into a charged residue (G156E, G159D, G164D, and G181R), with the first three clustered within a short stretch (positions 156–164 in OX20-8 allele). More interestingly, G159 and G164 appear to be part of a G-loop (GxGxxG) (Figure 2B, underlined) known to be essential for ATP-binding by protein kinases, hence their catalytic activities [28]. Moreover, the G-loop has also been reported to participate in dimerization of protein kinases, especially if the phosphorylation of the serine, threonine, or tyrosine residues sandwiched by the 2nd and 3rd G, which is a tyrosine (Y) in the case of QL-OX (Figure 2B), are realized through autophosphorylation [29,30]. Intriguingly, this G-loop motif is absent in QL18-PI, though we caution against interpreting this as QL18-PI lacking protein kinase activity, as many similar protein kinases of plant origin (e.g., NP_567053 of Arabidopsis and ABA94280 of rice) lack an obvious G-loop.

Nevertheless, these findings hint at a fascinating explanation for the failure of ZYE-QL-OX4 and ZYE-OX4-CS to infect soybean systemically. Note the 147-aa OX4 partial fragment was unlikely to have acted as a functional protein kinase. Rather, it is more plausible that this protein fragment could have formed heterodimers with the endogenous QL18-OX protein, thereby disrupting the function of the latter. This nonfunctional dimer could in turn have triggered a strong, intracellular hypersensitive defense response (also known as micro HR) that restricted the viruses in infected cells or local leaves. We hasten to note that the G-loop by itself was apparently insufficient to elicit this defense response, as evidenced by the infection outcome of ZYE-QL18-OX4d2 (Figure 3A,B). Furthermore, other mutations outside the G-loop apparently also enabled the evasion of this defense.

### 3.6. A Single Mutation Restores Mosaic Symptoms in Soybeans Overexpressing OX4, and Abolishes Apical Necrosis in Those Overexpressing PI4

**Figure 5:**
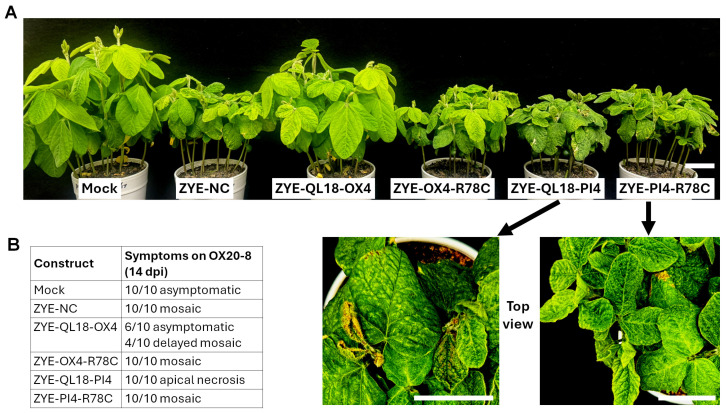
Symptoms of OX20-8 seedlings inoculated with ZYE constructs containing a single R78C mutation in OX4 and PI4 inserts. Scale bars = 5 cm.

With the identification of eleven different aa-changing mutations within the OX4 protein fragment expressed from the ZYE-QL18-OX4 and ZYE-OX4-CS constructs, we next wanted to determine whether such mutations alter viral symptoms when introduced into ZYE-QL18-OX4. To this end, we selected the R170C mutation in Figure 4C and Figure 2B. Note that R170C in the full-length QL18-OX protein corresponds to R78C within the 147-aa OX4 protein fragment (Figure 4C, bottom row). Since this particular aa residue was shared by QL18-OX and QL18-PI, we introduced the same R78C change into ZYE-QL18-4OX and ZYE-QL18-4PI, resulting in the mutant constructs ZYE-OX4-R78C and ZYE-PI4-R78C. When delivered into OX20-8 seedlings, ZYE-OX4-R78C, with just one aa difference from ZYE-QL18-OX4, was now fully competent at infecting soybean systemically, causing mosaic symptoms in 100% of inoculated plants (Figure 5A,B). Surprisingly, ZYE-PI4-R78C with the same mutation no longer elicited apical necrosis either. Instead, it caused all plants to develop mosaic symptoms similar to the ZYE-NC control (Figure 5A,B). Thus, the single R78C mutation abolished the unique symptoms associated with the virus-mediated expression of both OX4 and PI4 protein fragments.

### 3.7. A Spectrum of Symptoms Elicited by OX4-PI4 Chimeras in Three Different Soybean Accessions

**Figure 6:**
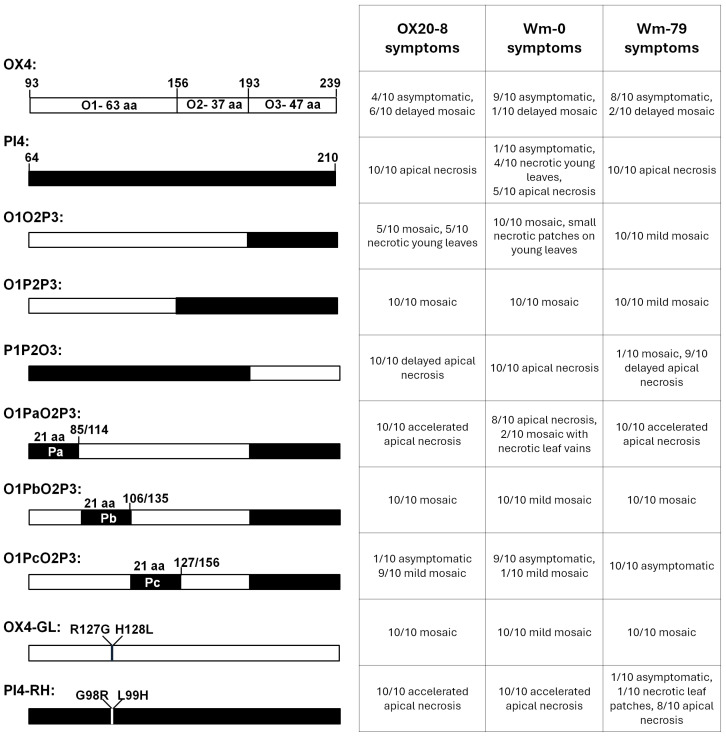
Infection outcomes of ZYE constructs containing chimeric OX4-PI4 inserts in OX20-8, Wm-0, and Wm-79 seedlings.

We next wished to resolve what caused the dramatic symptom differences in soybeans receiving ZYE-QL18-OX4 and ZYE-QL18-PI4 viruses. To this end, we divided OX4 into three sections: a 63-aa O1 section that is prior to the conserved protein kinase domain (GenBank ID cd14066), a 37-aa O2 section, and a 47-aa O3 section corresponding to the portion shared by OX4 and OX2 fragments (Figure 6, also see Figure 2A). Substituting various combinations of O1, O2, and O3 with their PI4 counterparts yielded three chimeric constructs: O1O2P3, O1P2P3, and P1P2O3 (Figure 6). Testing these constructs in OX20-8 soybeans revealed that all of them caused systemic symptoms, confirming that expression of the intact OX4 was needed to exclude ZYE-QL18-OX4 from systemic leaves of some plants.

By contrast, the systemic necrosis caused by PI4 expression appeared to entail additive actions of different PI4 sections. This is because the 100-aa P1P2 and the 47-aa P3 were by themselves capable of eliciting delayed necrosis in at least some of the inoculated plants (Figure 6). We next constructed three more chimeric constructs using O1O2P3 as the backbone, but substituting three equal subsections of O1 consecutively with their PI4 counterparts (Pa, Pb, Pc). Soybean (OX20-8) infections with these three chimeras revealed that co-existence of the N-terminal 21 aa (Pa) and C-terminal 47 aa (P3) caused soybeans to develop accelerated apical necrosis compared to PI4 itself. These results mapped the necrosis-inducing domains of PI4 to Pa and P3 regions. Conversely, Pb and Pc subsections appeared to repress or delay the necrosis induced by P3 (Figure 6).

Within the Pb subsection (aa positions 114–134 of QL18-OX, 85–105 of QL18-PI), OX4 and PI4 differed by just two aa residues—RH versus GL (Figure 2B). We thus wondered if exchanging these two aa residues might alter the symptoms elicited by OX4 or PI4. As expected, substituting RH in OX4 with GL overcame the partial failure of systemic infection, leading to mosaic symptoms in all plants. Surprisingly, substituting GL of PI4 with RH exacerbated PI4-mediated apical necrosis. Therefore, these two aa residues appear to be critical for the characteristic infection outcomes of both ZYE-QL18-OX4 and ZYE-QL18-PI4 constructs.

### 3.8. OX4-Mediated Partial Failure of Systemic Spread and PI4-Mediated Apical Necrosis May Reflect Varying Strengths of the Same Defense Response

To further interrogate the underlying mechanism of the symptom differences caused by virus-mediated expression of OX4 and PI4, we next used this set of chimeric constructs to infect two more soybean accessions: Williams (Wm-0 hereafter) and Williams 79 (Wm-79). Note that the QL18 alleles in Wm-0 and Wm-79 are identical to that of OX20-8 at the aa level, despite two differences at the nucleotide level (our unpublished results). The infection outcomes were highly similar to OX20-8 seedlings, but with a few notable differences. First, while PI4 caused uniform apical necrosis in both OX20-8 and Wm-79, it caused more variable symptoms in Wm-0, with a small fraction of seedlings being asymptomatic. Second, while P3 as part of the O1O2P3 chimera still elicited some necrosis in OX20-8 and Wm-0, it failed to do so in Wm-79. Most strikingly, while the O1PcO2P3 chimera elicited mosaic symptoms in most OX20-8 seedlings (9/10), it failed systemic infections in most (9/10) Wm-0 seedlings and all (10/10) Wm-79 seedlings.

Together these results suggest that the failed systemic infection might be manifestation of a strong HR that restricted viruses in single cells or inoculated leaves. Conversely, apical necrosis might have manifested the same defense response, but at somewhat weakened extents, permitting the viruses to spread systemically while continuing to trigger HR along the way, causing necrosis in apical tissues. If this is correct, then accelerated necrosis associated with O1PaO2P3 and PI4-RH might reflect an intensified systemic HR that nevertheless fell short of restricting the viruses in inoculated leaves, whereas the delayed or partial necrosis associated with P1P2O3 might reflect a weakening of PI4-triggered systemic HR. These findings might also suggest that QL18-OX is a key defense component of soybean, and whose perturbation can be sensed by an as-yet-unidentified resistance (R) protein to initiate a defense cascade that entailed HR.

## 4. Conclusions

With the current study we upgraded a CPSMV-based vector system that can be used to interrogate functions of soybean genes through VIGS or virus-mediated expression of proteins or protein fragments. We further used this PENTIN-ZY vector system to examine the function of QL18, a putative soybean protein kinase, and found that virus-mediated expression of two allelic partial fragments of QL18, derived from two different soybean accessions (OX20-8 and PI427105B), elicited drastically different symptoms in soybean. Overall, our results suggest that the OX20-8-derived partial fragment, designated OX4, induced a strong HR-based defense response that restricted the corresponding vector virus inside the inoculated leaves, or even cells of these leaves, of most soybean seedlings. On the other hand, the PI 427105B-derived partial fragment, PI4, likely triggered a somewhat weaker HR. As a result, the PI4-expressing virus was still able to spread systemically, but continued to trigger a systemic HR in the apical portion of the soybean seedlings invaded by the virus. Combined with the earlier finding that the PI allele of QL18 conferred a partial resistance to *P. sojae*, our results could suggest that an HR of moderate strength, capable of spreading to multiple cells, might be needed to subdue extracellular pathogens such as *P. sojae*. Alternatively, the OX20-8 allele of QL18, which is shared by many other soybean accessions, might be targeted by one or more *P. sojae* counter-defense effectors for inactivation. Conversely, the PI 427105B allele of QL18 could have evolved to evade such inactivation events. These possibilities await further investigations.

## Data Availability

All data have been described in the manuscript. Please contact F.Q. (qu.28@osu.edu) for any materials. The PENTIN and ZY constructs are being deposited in Addgene (https://www.addgene.org/ (accessible after 1 September 2026)) for community sharing.

## Supplementary Materials

The following supporting information can be downloaded at: https://www.mdpi.com/article/10.3390/v18040419/s1, a Word document containing Table S1: Sequences of synthesized DNA fragments used in this study; Table S2: Sequences of deoxyribonucleotide primers used in this study.
