# Supplementary material for: Virus-Mediated Overexpression of Two Allelic Protein Fragments Elicits Drastically Different Responses in Soybean
Source: Viruses. 2026 Mar 29;18(4):419. doi: 10.3390/v18040419 (PMC13120491; doi:10.3390/v18040419)
Supplement: Supplementary file 1 [file viruses-18-00419-s001.zip › viruses-4228191-supplementary.pdf]

Table S1. Sequences of synthesized DNA fragments used in this study

Name	Sequence	Purpose/Note
PENTIN<br>(675 bp)	TTCAAAATCACTTTTTCTCTCTTAAGCTACACAAGTTTAACTAAAAGCCTAGAACGAAC<br>TTTTTATTGGACGCATTAAACTACAATATGAAGTTCTTTGCTGGGCAAACTGTTATGAAT<br>GTGCTGCAACATGTTTCCTCTCCCACCACCAATTTAAGGTTGTTATCTTATTGCAATCT<br>TAAAAAGGAAGAGGATGGTAAGATGATGCTGGCTATTAAGGAGCAGAGGCACCGA<br>CGGTTGTTGACACTGTCATATGGTGCTATGTGTTTTCAATTCTCAAATTCAGTAGGGGA<br>TGAGGTATCTTCTAACATTTGCAAGTAATGTCTTACATCAGTCAGAACACCAAAGAAAT<br>ATCTTTCCATGTGGATTGGTTTGATAGGGCATAGAAGTGGATGATGATGAGTTGATGTT<br>TGAAATATTTGATGCATTGCTGCGCACAAAGATTTCAAACTCAAAGGGCATGACGCA<br>CTTATACAGCTGGATGCGTGGAGTTTATCTCAGCACATTCAAAGTAGAGGTGCAGTG<br>TGATGATTACAACTCAAATCTGCTGGAGAAGGATTTGGCTGGAGAAGCTCAGGGTCT<br>TTCACAGTTCGTTTCAGGACTTGCTGACTGGATTCCCAGTCGTGTAAAGACCTTGGC<br>AGGATATGCCGCTGAGGGCATAATTGAGGCCTTTAAG	Introducing the 5th intron into RNA1 cDNA. The 82-nt intron (red font) is the 18th intron of At3G43920.2.<br>Underlined are nts of AflII and BbvCI sites used to clone this fragment.
ZYB-NbTOR<br>(786 bp)	GCTTCTCAGTCCGCAATATTTGAGATATTCTATCGGCTTGATAATTCATGGAAAAGTGC<br>TGGAAATGTTTTAATGCCACCATTGTTGGTTGGGAATCCCAGGCTTGACATCAAAGG<br>TCGTGCCGCAGCAGCTGCATAGTACTATACTTTCGAAAGTTGAGGTCATCAGTGATC<br>CTTTGAGTGATGGAGAGAATGAGCACTACGAGGTTGGGACGGACATGCATAAGCAG<br>CTTAAAAGCCATCAGGTTAATGATGGTAGATTGCGTACCGCTGGTGAGGCTTCTCAA<br>CGAAGCACTAAAGAGGATTGGGCAGAGTGGATGAGGCATTTCAGCATTGAACTTCT<br>GAAAGAATCACCTAGTCCAGCATTGCGAACTTGTGCAAGACTCGCTCAACTGCAGC<br>CTTTTGTTGGGCGAGAGTTGTTTGCTGCAGGTTTTGTTAGCTGCTGGTCACAACTTTC<br>GAACTATTTGCTTTAAATAAGCCTCATAAGTCTATGACCCCAATTTGGTATGCTCTAGC<br>AGAGGTAAGTTTGGTTTGCAATTTCTTTATTTTTGTTGTAAACATTGTAATTAGCGTATTG<br>TGTGGTAGTATATGTTATGCTCTTTTCACATTTCATCCATGTATTCCCAAGTCACTTTGA<br>CTCTAGGTAAGTGGATTAAGCCTTACATACCAGGCCGGTCTTGGGCAGCGACTGTT<br>AAATATGTCCAATCACTGAGGTATCATACGCCATTTAGTTGGTTGTGTTGATATCGTCTA<br>AACAGTTGGCGTTAACGCTTATGCATACATTCAG	Introducing new Bsp119I (TTCGAA) sites into FZ within 3' UTR of RNA2. Underlined nts depict Bsp119I and HpaI sites used for introducing the fragments. Note the original Bsp119I site was abolished by point mutations (no aa change). Two new Bsp119I sites (painted yellow) were introduced 11 nt after the stop codon (painted red). Blue nts denote a 300-nt insert replaceable by other inserts.
ZYB-NbPDS<br>(347 bp)	CTGCATAGTACTATACTTTCGAACAAGGTGTGCCTGATAGGGTGACAGATGAGGTGT<br>TCATTGCCATGTCAAAGGCACTTAACTTCATAAACCCTGACGAGCTTTCGATGCAGT<br>GCATTTTGATTGCTTTGAACAGATTTCTTCAGGAGAAACATGGTTCAAAAATGGCCTTT<br>TTAGATGGTAACCCTCCTGAGAGACTTTGCATGCCGATTGTGGAACATATTGAGTCAA<br>AAGGTGGCCAAGTCAGACTAAACTCACGAATAAAAAAGATCGAGCTGAATGAGGAT<br>GGAAGTGTCAAATGTTTTATACTGAATAATGGCAGTTTCGAACTATTTGCTTTAAATAAG	For introducing an NbPDS fragment into ZY at the Bsp119I site.<br>The NbPDS-derived fragment is in sense orientation.
ZYB-<br>NbPDSrc<br>(347 bp)	CTGCATAGTACTATACTTTCGAAACTGCCATTATTCAGTATAAAACATTTGACACTTCC<br>ATCCTCATTCAGCTCGATCTTTTTTATTCGTGAGTTTAGTCTGACTTGGCCACCTTTTG<br>ACTCAATATGTTCCACAATCGGCATGCAAAGTCTCTCAGGAGGGTTACCATCTAAAA<br>AGGCCATTTTTGAACCATGTTTCTCCTGAAGAAATCTGTTCAAAGCAATCAAAATGCA<br>CTGCATCGAAAGCTCGTCAGGGTTTATGAAGTTAAGTGCCTTTGACATGGCAATGAA<br>CACCTCATCTGTCACCCTATCAGGCACACCTTGTTCGAACTATTTGCTTTAAATAAG	For introducing a reverse-complemented (rc) NbPDS fragment into ZY at the Bsp119I site.
ZYE-QL18-<br>OX4	CTATCTCAGATACGATGCACGTGTCTCCTTCTCCTGTCCAGAGTCTTGCTTCGGCAG<br>TGCCCAGTGCAATGAGCAGGAGTAAAGCTGCACCATTTTCACAACTGGAAGTGGTG<br>CCGAACGCCACTAGACATGTTCCTGCACGATTATTCACCTGGGCTGAGCTTAAAGC<br>AGCCACCAACAATTTCTCACCTCACAACCGGATTCGTCATTTTGGAACCTTTGGCTT<br>TGGGTACAGAGGCAAACTCGTTGATGGTCGTGAGGTCGCAATCGTTAAGGGAACC<br>CAGTTTGGTAAGTCTGAATTTGCCATCTTTTCCCGTTTAGACCATAGGAACTTGGTTG<br>GGCTTGTTGGGTACTGTGAAAACAGAGATGAAAGGCTGTCAGTTTACGAGTATATGA<br>AGAATGGGTTGTTGCGTGATTGCTTGCATGATAAGAACAATGTGGACAAGGATAGCA<br>GTGTGTTGAATCACGTGACGTTCGATACCACACA	Introducing the OX4 insert into ZYE
ZYE-QL18-<br>PI4	CTATCTCAGATACGATGCACGTGTCTCCTTCTCCTGTCCATAGTCTTGCTTCGGCAGT<br>GGCCAATGCAATGAGCAGCAGTAGTGCTGCACCATTTTCACAACTGGAAGTGGTG<br>CCCAACGCCACTGGACTTGTTCCTGCACGATTATTCACCTGGTCTGAGCTTAAAGA<br>AGCCACCTACAATTTCTCACCTCACAACCGGATTCGTCATTTTGGAAGCTTTTTCTTT<br>GTGTACAGAGGCAAACTCGTTGATGGTCGTGAGGTCGCAATCGTTAAGGAAACCCC<br>GTTTGGTAAGTCTGAATTTGCCATCTTTTCCCGTTTAGACCATAGGAACTTGGTTGGG<br>CTTGTTGGGCACTGTGAAAACAGAGATGAAAGGCTGTCAGTGTATGAGTATATGAAG<br>AATGGGTCATTGCATGATTGCTTGCAAGATAAGAACAATGTGGACAAGGATTGCAGT<br>GTGTTGAATCACGTGACGTTCGATACCACACA	Introducing PI4 insert into ZYE

Table S2. Sequences of deoxyribonucleotide primers used in this study

Name	Sequence	Purpose/Note
NbACT-F2	CAGCCACACTGTCCCAATTTATGAG	RT-PCR detection of NbACT mRNA
NbACT-R	CACCTTAATTTTCATACTGCTTGGA	RT-PCR detection of NbACT mRNA
NbPDS-450F	TGCATGGAAAGATGATGATGGAGAT	RT-PCR detection of NbPDS mRNA
NbPDS-979R	GTCTCTCAGGAGGGTTACCATCTAA	RT-PCR detection of NbPDS mRNA
GmACT4-F2	TCAACCACTCGTCTGCGATAATG	RT-PCR detection of soybean actin 4 mRNA
GmACT4-R2	CCAGCTTTTCCTTTACATCCCTTA	RT-PCR detection of soybean actin 4 mRNA
P35S-F2	CACAATCCCACTATCCTTCGCAAGA	Sequencing the CPSMV RNA1 portion with the 5th intron
CPSMR2-1159F	GAGATCTGGCGATGATGCATGTGAAT	RT-PCR detection of CPSMV RNA2 and ZYE inserts
CPSMR2-1385F	GAGAATCTCATCTACCAAAGCTAT	RT-PCR detection of CPSMV RNA2 and ZYE inserts
CPSMR2-1679R	GAGAACTGTACCTGCAACTAATG	RT-PCR detection of CPSMV RNA2. Sequencing ZYE inserts
CPSMR2-3686R	GCACACTTTTATAAATAATTCTAAAC	Sequencing ZYB inserts
